# Severe neonatal hyperbilirubinemia induces temporal and occipital lobe seizures

**DOI:** 10.1371/journal.pone.0197113

**Published:** 2018-05-11

**Authors:** Lian Zhang

**Affiliations:** 1 Department of Neonatology, Shenzhen City Baoan District Women and Children’s Hospital, Shenzhen, People’s Republic of China; 2 Department of Neonatology, Guangzhou Women and Children's Medical Center, Guangzhou, People’s Republic of China; University of Electronic Science and Technology of China, CHINA

## Abstract

To examine the origin of seizures induced by severe neonatal hyperbilirubinemia, The EEG characteristics of seizures were analyzed in newborns with and without severe neonatal hyperbilirubinemia. Fisher’s exact test was used to determine the specificity. In total, 931 patients had a total serum bilirubin (TSB) level of 340–425 μmol/L, only 2 of whom had seizures. Compared to patients with hyperbilirubinemia and a TSB level of 340–425 μmol/L, those with a TSB level >425 μmol/L had a significant risk of seizure (OR = 213.2, 95% CI = 113.0–405.8, P<0.001). Of all 28 patients with severe hyperbilirubinemia and seizure, 26 had seizures that originated in the temporal and/or occipital lobe. In seizure patients without severe hyperbilirubinemia, origination in the temporal/occipital and other lobes occurred in 19 and 117 cases, respectively. Compared to the risk of seizure origination in the temporal and/or occipital lobe in other diseases, the risk in patients with severe hyperbilirubinemia was increased by approximately 80 times (OR = 80.1, 95% CI = 28.3–226.4, P<0.001). Severe neonatal hyperbilirubinemia can selectively induce temporal and occipital lobe seizures. This is the first report of a new syndrome with the same etiology and electrophysiological features as epilepsy.

## Introduction

Severe neonatal hyperbilirubinemia can lead to brain damage, including bilirubin encephalopathy and kernicterus. These long-term outcomes are not common nowadays due to the availability of timely and effective interventions for hyperbilirubinemia, for example, exchange transfusion, maternal rhesus immunoglobulin prophylaxis and phototherapy[1]. However, kernicterus can occur in healthy near-term and term infants without hemolytic disease or risk factors for hemolysis[2]. Some of the associated risk factors for severe hyperbilirubinemia are: jaundice in the first day of life or before discharge, having a compatriot who had jaundice underwent phototherapy, late preterm with the gestational age of 35–36 weeks, Asian race, the presence of infant bruising or cephalhematoma, rhesus and ABO incompatibility, as well as glucose-6-phosphate dehydrogenase (G6PD) deficiency[1].

Guidelines for managing jaundice have recently been implemented, and hence the incidence of total serum bilirubin (TSB) of more than 25 mg/dL has decreased, whereas the trends for TSB greater than 30 mg/dL remained relatively stable from 2007–2012 in California (trend −0.37 per 100,000 live births per year, 95% CI = −1.23 to 0.49, P = 0.30 and R^2^ = 0.26)[3] along with the incidence of the newborns who underwent exchange transfusion. As a result, the resurgence of severe neonatal hyperbilirubinemia and kernicterus is of grave concern[4].

The majority of the neurological features of severe hyperbilirubinemia have been reported. Since the classic vulnerable location of severe hyperbilirubinemia is the basal ganglia (BG), the corresponding consequence of hearing loss frequently occurs[5]. However, if the concentration of serum unconjugated bilirubin is increased to an extremely high level, other locations in the brain, such as the cortex, could be involved. Previously, Gürses et al[6] reported that hyperbilirubinemia (16.2–33 mg/dL) affected cerebrocortical electrical activity. It is unknown whether much higher cortical electrical activity could be induced by severe hyperbilirubinemia and ultimately trigger seizures because the characteristics of seizures caused by severe hyperbilirubinemia have not been previously studied. The present manuscript describes the origination of this type of seizure and analyzes the possible neural projections between the BG and the involved cortex.

## Materials and methods

### Patients

The study included all newborn infants with seizures admitted to the Guangzhou Women and Children’s Medical Center from January 2012 to January 2016. Seizure patients with severe hyperbilirubinemia were separately documented and analyzed.

Similar to previous research[1], severe hyperbilirubinemia was defined as infants aged less than 60 days with unconjugated hyperbilirubinemia and a peak TSB level of more than 425 μmol/L or who had undergone a neonatal exchange transfusion, or both. A bilirubin level of more than 425 μmol/L was chosen to define severe hyperbilirubinemia because of the high risk of kernicterus at this level[7]. Accordingly, the Canadian Pediatric Society recommends considering an exchange transfusion at this level in healthy term infants without risk factors[8]. Full channel video EEG, brain MRI and brainstem auditory evoked potential (BEAP) were conducted on all patients with severe hyperbilirubinemia.

Hyperbilirubinemia was defined as a TSB level above the phototherapy limit recommended by the American Academy of Pediatrics[9]. The specific inclusion criteria were a gestational age of 37–42 weeks, no anti-convulsion drug history, and no hypoxic-ischemic encephalopathy history or other disorders that may affect brain function. The general examination included a complete blood count, reticulocyte count, blood gas analysis, albumin levels, blood type, Coombs test, blood culture, serum electrolytes, glucose-6-phosphate dehydrogenase (G-6-PD) measurement and head ultrasound. After examination, children with sepsis, intracranial hemorrhage or metabolic diseases were excluded. TSB was measured by the oxidizing method (Maccura, Sichuan, China) on a Hitachi 7600 auto analyzer[10], and erythrocyte G-6-PD deficiency was measured by the quantitative G6PD/6PGD ratio method (Micky, Guangzhou, China)[11]. The children were treated with phototherapy and/or exchange transfusion in the hospital. The phototherapy and exchange transfusion standard was consistent with the 2004 guidelines and updates of the American Academy of Pediatrics[9]. According to the local hospital policy, continuous video EEG was applied to all term newborns who suffered from hyperbilirubinemia with a maximum TSB level of greater than 340 μmol/L.

Shortly after the diagnosis of severe hyperbilirubinemia, the newborns underwent MR examination. All brain MRI scans were performed on 1.5T whole-body MRI systems (Achieva, Philips Healthcare), with the subjects under sedation. Axial, sagittal and coronal T1-weighted spin-echo (TR: 590 ms; TE: 15 ms), T2-weighted turbo spin-echo (TR: 3723 ms; TE: 100 ms) and axial FLAIR (TR: 6000 ms; TE: 100 ms) images were obtained. The sequence were turbo spin echo (TSE) and fluid attenuated inversion recovery(FLAIR) in T2-weighted imaging, turbo inversion recovery(TIR) in T1-weighted imaging. The Signal-to-noise ratios (SNR) of basal ganglia is about 18–27 in T2-weighted imaging, 30–60 in T1-weighted imaging and 10–15 in T2-FLAIR imaging respectively. The SNR and Matrix were high enough to recognize abnormal increased signal from normal.

The Ethics Committee of Guangzhou Women and Children’s Medical Center approved this clinical study protocol (approval number 2017082501). General informed consent was given by parents upon admission for all patients’ biological samples to be used for medical research. Electronic signed consent from each infant’s legal guardians was obtained after informed consent.

### EEG settings

EEG monitoring was conducted by an experienced EEG technician with a bedside polyparameter digital EEG device (Nicoletone Thirty-two Channel video Monitorone, VIASYS Healthcare, U.S.A.). As described previously[12], a prewired EEG cap containing 11 recording electrodes (P3 and P4 were added) was used for monitoring. The recording electrodes, which were placed according to the modified 10–20 system for neonates, consisted of Fp1/Fp2/C3/ Cz/C4/P3/P4/O1/O2/T3/T4, along with a ground electrode. The average reference (AR) is chosen to be the reference electrode. AR is a virtual electrode which calculated by the computer from the average of all recording electrodes plus 20 Ω impedance. AR has the average voltage of the above 11 recording electrodes plus a 20 Ω impedance. Polyparameters were obtained from a pair of electromyogram electrodes, bilateral eye electrodes, a pair of respiratory electrodes and a pair of electrocardiographic electrodes. All recordings were synchronized. The EEG noise was processed by the high frequency filter of 70 Hz and low frequency filter of 0.5 Hz (equals to time constant of 0.3 second) respectively.

Two additional expert electroencephalographers evaluated the EEG and synchronized video information to the locate seizure origin and document the seizure onset as well as the evolution, termination and duration of the seizures. The EEG results were considered the final standard. An electrographic seizure was defined as a sudden, repetitive, evolving, and stereotyped ictal pattern having a clear beginning, middle and end with an amplitude of ≥2μV and a minimum duration of 10 s[13]. Unless otherwise indicated, all seizures presented in this paper are termed as electrographic seizures. Individual seizures separated by at least 10 s were considered separate events.

During the conventional EEG recording an amplitude distribution chart was obtained synchronously which extracts the amplitude information. The amplitude changes of the recording area indicate the highest to lowest amplitude with red to blue gradient color scale. Intuitively, amplitude ardor frequency of the EEG will change during the evolution of a seizure. Amplitude distribution charts was created to localize the most affected area during a seizure.

### Statistical analysis

Fisher’s exact test was performed using R 3.2.2 statistical package for Windows (https://www.rproject.org/about.html). The relationships were expressed in fourfold tables.

## Results

From January 2012 to January 2016, a total of 164 term newborns with video EEG confirmed seizures were admitted to the Guangzhou Women and Children’s Medical Center. Among the confirmed cases, 28 newborns had severe hyperbilirubinemia. The clinical features of the 28 cases are documented in Table 1. MRI findings were not included because all patients had abnormal increased signal intensity BG imaging on T1-weighted imaging. None of the patients had putamenal, thalamic, subthalamic or hippocampal involvement on MR images.

**Table 1:** Clinical features of the patients with seizure and severe hyperbilirubinemia.

Patient	gender	Gestation (weeks+days)	Age of the first seizure (day)	Underlying reason of jaundice	Max TSB(μmol/L)	BAEP(grade)	Seizure origination	Seizure frequency (times per hour)
**1**	female	37+6	3	G-6PD deficiency	489.5	4	temporal, occipital	6
**2**	female	38+1	4	unknown	541.3	5	temporal, occipital	3
**3**	male	38+2	2	G-6PD deficiency	674.2	5	occipital	22
**4**	female	41+5	9	G-6PD deficiency	493.6	5	central, frontal	10
**5**	male	40+2	4	BI	524.1	4	temporal, occipital	14
**6**	female	38+7	7	unknown	470.1	5	occipital	26
**7**	male	37	5	BI	614.9	4	temporal, occipital	3
**8**	male	37+3	8	sepsis	712	5	temporal, occipital	11
**9**	male	40	5	G-6PD deficiency	996.2	3	occipital	35
**10**	male	37+5	3	G-6PD deficiency	650.4	5	temporal, occipital	2
**11**	female	38	4	G-6PD deficiency	645.3	5	temporal, occipital	42
**12**	male	39+2	2	G-6PD deficiency	640.2	5	occipital	6
**13**	male	40+1	2	G-6PD deficiency	616.9	5	temporal, occipital	17
**14**	male	40+5	3	G-6PD deficiency	600	3	temporal, occipital	20
**15**	female	38+2	3	BI	591.2	5	temporal	5
**16**	female	37+2	1	unknown	483.9	5	temporal, occipital	33
**17**	female	37	3	unknown	532.2	5	temporal, occipital	24
**18**	female	37+5	1	unknown	700	5	temporal, occipital	15
**19**	male	37+7	6	G-6PD deficiency	547.2	4	temporal, occipital	10
**21**	male	38+4	3	G-6PD deficiency	687.6	5	temporal, occipital	8
**22**	female	39	2	BI	475.2	5	occipital	4
**23**	male	38+6	8	unknown	622.3	3	central	14
**24**	female	37+1	5	G-6PD deficiency	741.5	5	temporal, occipital	10
**25**	female	38	5	unknown	644	5	temporal, occipital	25
**26**	male	38+5	3	unknown	712.3	3	temporal, occipital	14
**27**	male	37+4	4	unknown	556.1	4	temporal	18
**28**	male	40+3	10	G-6PD deficiency	490.4	5	temporal, occipital	27

BI: Blood group incompatibility; BAEP: brain-stem auditory evoked potential; Grade 1–5 represented normal, mild abnormal, moderate abnormal, severe abnormal and no response respectively; The gray shade of patient 4 and 23 indicate the exceptional origination of seizure.

During the same period, long-term video EEG was applied to all 1,020 neonates with TSB levels greater than 340 μmol/L to detect seizures. The results are shown in Table 2.

**Table 2:** The relationship of seizure and severe hyperbilirubinemia.

	seizure detected	seizure not detected	OR (95% CI)	P
Hyperbilirubinemia with TSB 340–425μmol/L	2	929	1.00	<0.001
Severe hyperbilirubinemia (TSB>425μmol/L)	28	61	213.2 (113.0–405.8)	

To describe the relationship between severe hyperbilirubinemia and seizures, all patients with a TSB level greater than 340 μmol/L were further analyzed. Fisher’s exact test was used to examine the specificity of seizure detection for patients with severe hyperbilirubinemia. As shown in Table 2, of all patients with severe hyperbilirubinemia, 28 had seizures, and 61 did not have seizures. Of the 931 patients with a TSB level of 340–425 μmol/l, only 2 had seizures. Compared to the patients with hyperbilirubinemia and a TSB level of 340–425 μmol/L, those with a level >425 μmol/L had a significant risk of seizure (OR = 213.2, 95% CI = 113.0–405.8, P<0.001).

As shown briefly in Table 1, most of the seizure patients with severe hyperbilirubinemia exhibited origination in the temporal and/or occipital lobe. Fisher’s exact test was used to examine the specificity of temporal and/or occipital seizures. As shown in Table 3, of all 28 patients with severe hyperbilirubinemia and seizures, 26 exhibited origination in the temporal and/or occipital lobe, whereas only 2 exhibited origination in other locations. In seizure patients without severe hyperbilirubinemia, 19 and 117 showed origination of seizures in the temporal/occipital lobe and other lobes, respectively. Compared to other diseases, severe hyperbilirubinemia increased the risk of origination in the temporal and/or occipital lobe by approximately 80 times (OR = 80.1, 95% CI = 28.3–226.4, P<0.001).

**Table 3:** The relationship of temporal and/or occipital lobe seizure and severe hyperbilirubinemia.

	seizure with temporal and /or occipital origination	seizure with other origination	OR (95% CI)	P
Seizure without severe hyperbilirubinemia	19	117	1.00	<0.001
Seizure with severe hyperbilirubinemia	26	2	80.1 (28.3–226.4)	

**Fig 1:**
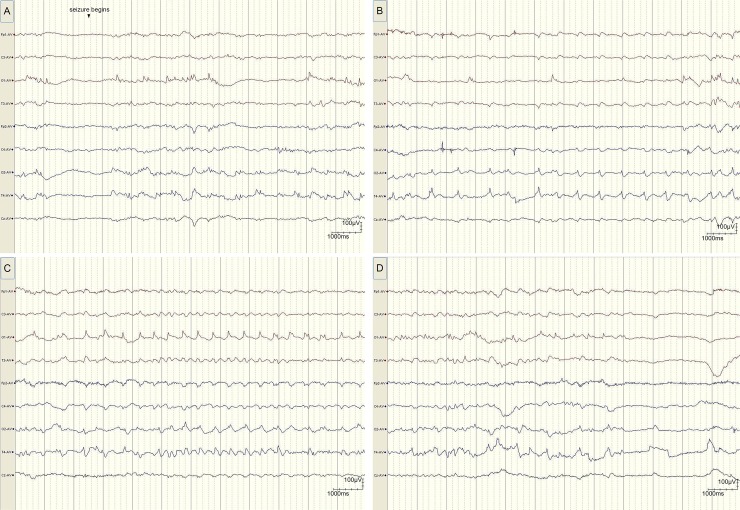
A typical time course of seizure evolution process at day 6 (patient number 21). “Seizure begins” indicated the initiation of seizure. A(0-12s of seizure) displayed a typical seizure which originated from occipital and temporal lobe(O_1_,O_2_,T_4_); spikes evolved into shape waves rhythm with broad base emphasized on the right hemisphere (B, 18-29s of seizure, O_2_,T_4_); Left hemisphere get involved mainly on the occipital lobe (C, 30-41s of seizure,O1); seizure ended with temporary voltage depression (D, 45-56s of seizure).

**Fig 2:**
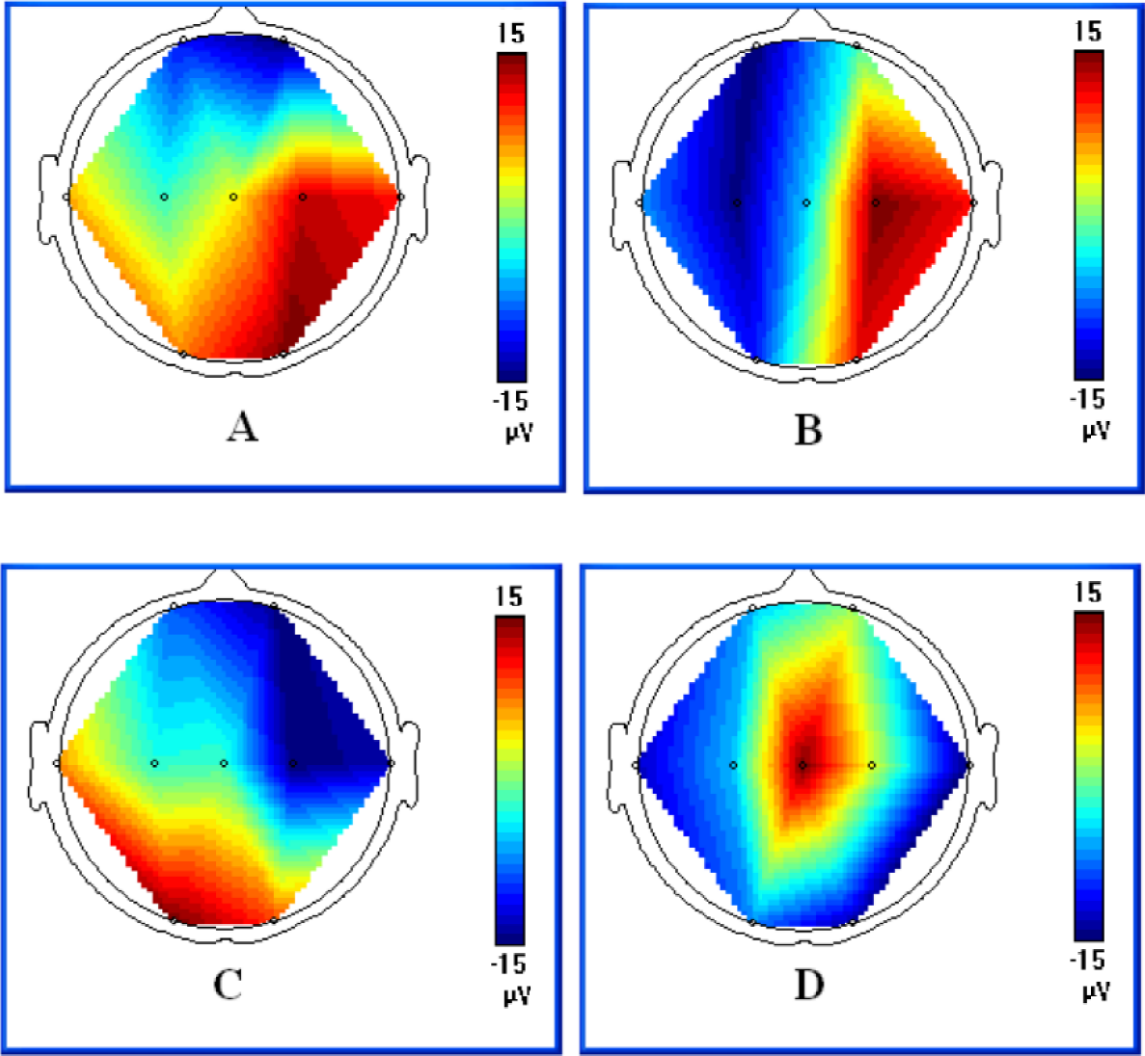
Amplitude distribution charts of the same seizure in Fig 1. The charts show the amplitude changes of the recording area which indicates the highest to lowest amplitude with red to blue gradient color scale. A displayed the highest amplitude distribution on bilateral occipital and right temporal lobes as the initial of the seizure (O1, O2, T4, corresponding to A of Fig 1); B showed a migration to the right hemisphere emphasized on occipital and temporal lobes (O2, T4, corresponding to B of Fig 1); C indicated left occipital dominated again (O1, corresponding to C of Fig 1); Both temporal gained the lowest amplitude when the seizure ended (corresponding to D in Fig 1) in D.

**Fig 3:**
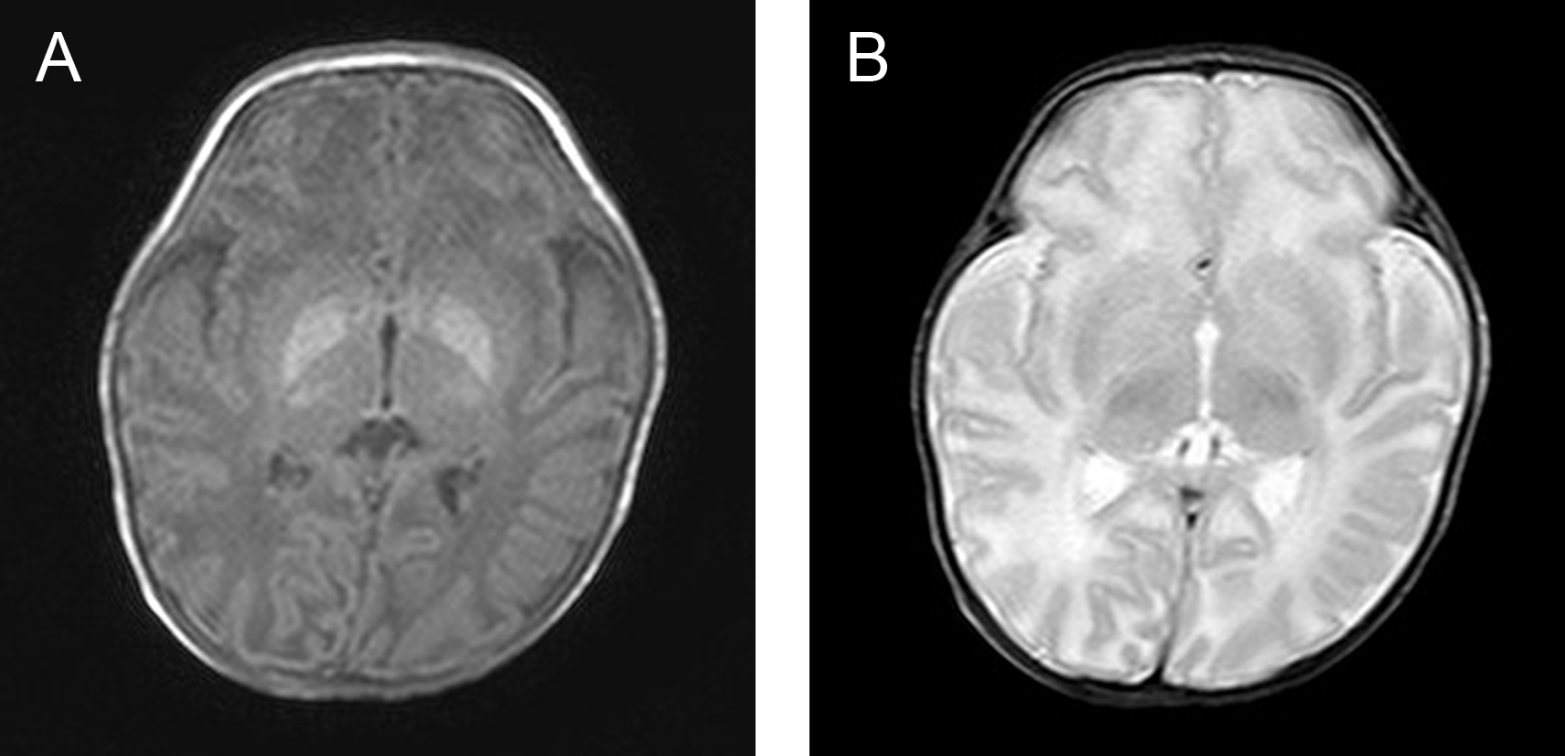
T1 and T2 weighted MR imaging at day 7 (patient number 21). A: There was bilateral, symmetric, abnormal increased signal intensity in the GP on T1-weighted imaging. B: The above-mentioned lesions were not apparent on T2-weighted images.

Typical EEG traces, amplitude distribution charts and MRI findings of a same patient are shown in Figs 1,2 and 3. Fig 1 recorded a typical time course of seizure evolution process at day 6 (patient number 21). Fig 2 displayed the sequential amplitude changes during seizure which indicated the highest to lowest amplitude with red to blue gradient color scale. Fig 3 shown T1 and T2 weighted MR imaging at day 7 of the same patient, which indicated the increased signal intensity in the GP on T1-weighted imaging and negative findings on T2-weighted images.

## Discussion

The neurological symptoms of severe hyperbilirubinemia vary greatly. Although seizure is not a common complication during the neonatal period, it can be observed in severely icteric infants. The present study clearly indicated that the patients with a level >425 μmol/L had a significant higher risk of seizure, compared to those with a TSB level of 340–425 μmol/L. The result might imply the necessity of routine video EEG application to all newborns with severe hyperbilirubinemia.

MRI is the imaging modality of choice. The characteristic abnormalities in kernicterus are bilateral, symmetric, high-intensity signals in areas that correspond to the globus pallidus (GP), seen in both T1- and T2-weighted images, representing pathological deposition of unconjugated bilirubin. However, most previous studies have relied on images obtained when infants were in the intermediate or late phase of injury, months to years after the onset of acute encephalopathy, and all these studies have placed emphasis on T2-weighted hyperintense images of the GP[14]. However, in the present study, which was conducted during the acute period of progression, hyperintense on T1-weighted MR imaging was observed, without any abnormality on T2-weighted scanning. The finding was consistent with other previous researchers and was believed to be the characteristic change of acute kernicterus[15].

In recent years, the infinity zero reference using the reference electrode standard technique (REST) has been increasingly applied, while the average reference (AR) was generally advocated as the best available reference option in previous classical EEG studies[16,17]. Unfortunately, my EEG platform doesn't support REST, so that the AR becomes an alternative choice. Theoretically the average reference is a virtual electrode which calculated by the computer from the average of all recording electrodes plus 20 Ω impedance. A comparative study[18]designed the EEG experiments on adults and performed a direct comparison between the influences of REST and AR on EEG-revealed brain activity features for three typical brain behavior states. The analysis results revealed that typical EEG features might be more clearly presented by applying the REST reference than by applying AR only when using a 64-channel recording. Actually, the high density electrodes recording situation of 64 or 128-channel unlikely occurs clinically in neonates' EEG recordings (mainly 9–12 channels) because of the head circumstance. The results also showed that there is no significant difference in the alpha-wave-blocking effect during the eyes-open state compared with the eyes-closed state for both REST and AR references and there was clear frontal EEG asymmetry during the resting state, and the degree of lateralization under REST was higher than that under AR. The study suggested that AR which was performed in my study was better or at least not worse than REST in many situations. In the present study, the AR reference is relatively a good choice for removing physical artifacts such as heart beats and body movements, especially for neonates' long term recordings with limited recording electrodes.

The present study summarized the novel electrophysiological characteristics of seizure onset in a very specific group of patients with severe hyperbilirubinemia. Here, I conclude that severe neonatal hyperbilirubinemia induces temporal and occipital lobe seizures. AlOtaibi et al[19] reported 12 newborns who suffered from severe hyperbilirubinemia and bilirubin encephalopathy, with only 5 having undergone EEG. The EEG results indicated multifocal spikes, generalized spikes and polyspikes, as well as discontinuous and intermittently asynchronous backgrounds. Unfortunately, the researchers did not identify electrographic seizure with video EEG, and as a result, the origination of the seizure was unclear. For this reason, I suggest that continuous video EEG, which is the gold standard for neonatal seizure diagnosis[20], should be conducted on all newborns with severe hyperbilirubinemia. Gürses at al[6] found a change in cerebrocortical electrical activity without a regional difference caused by moderate to severe hyperbilirubinemia (16.2–33 mg/dL). I assume that the bilirubin level in their study was not sufficiently increased to trigger seizures and reveal the differing vulnerability of the cortex.

Despite these previous findings, studies focused on the origination of neonatal seizures are lacking. Previously, I observed 876 individual seizures in 62 newborns[12], with all episodes confirmed by full-channel video EEG. Of all 876 seizures, 509 (58.1%) had a central origination, while 367 (41.9%) originated in other locations. None of the patients suffered from severe hyperbilirubinemia. This result illustrated that seizures of non-central origins were not an overwhelming phenomenon during the neonatal period, regardless of the underlying cause of the seizure. Therefore, it appears that temporal and occipital lobe seizures occur due to a specific cause, such as severe neonatal hyperbilirubinemia, and this phenomenon has been strongly confirmed by Fisher’s exact test in the present study. Few previous studies have described the specific features of seizures that accompany severe hyperbilirubinemia.

The mechanism of how severe hyperbilirubinemia, a disease typically caused by BG injury, induces temporal and occipital lobe seizure is unknown. The present finding indicates a possible neural projection between the BG and the temporal/occipital cortex, and substantial evidence suggests an inhibitory role of the BG during temporal lobe seizures.

Recently, interest in the role of the BG in epilepsy in adults has increased due to its prospective use as a deep brain stimulation target for treating seizures[21]. Both morphological and functional connections between the BG and cortex have been widely confirmed[22]. Based on the large body of experimental data, the BG nuclei have been proposed to be responsible for controlling cortical seizure activity. Epileptic activity is a highly integrated function of the brain that involves both cortical and subcortical structures, and seizures appear to result from abnormal organization or reorganization of cortical oscillating circuits. The BG and cortex interact in the processing of physiological and pathological cortical rhythms. Multiple studies have suggested an inhibitory role of the BG during temporal lobe seizures[23]. As a result of the feedback pathway to the cortex, BG may remotely impact the cortical oscillatory processes that are involved in the control of epileptic seizures. It appears that the BG does not generate specific epileptic EEG activity. Despite the absence of spikes, the BG participates in changing or reflects changes in the distribution of the ictal epileptic activity[24].

However, compared to the adult brain, several age-specific factors exist that are specific to the neonatal brain and lead to enhanced excitability and seizure generation[25]. Given that neuronal activity is critical for synaptogenesis and brain development, excitation predominates over inhibition in neuronal networks of the cerebral cortex during the neonatal period and the first few years of life[26]. Excitatory ion channels and transporters are expressed at levels that promote excitation, whereas inhibition is relatively underdeveloped compared to later in life. Hence, I suggest that the damaged BG of infants with severe neonatal hyperbilirubinemia are not able to inhibit the already elevated hyperactivity in the neonatal cortex, and as a result, seizures cannot be prevented. Two possibilities may explain the vulnerability of the temporal/occipital lobe. ①Severe neonatal hyperbilirubinemia directly increases the excitability of the temporal/occipital lobe. ②Connectivity between the BG and the temporal/occipital lobe facilitates epileptogenesis in the developing brain of preterm infants. Based on the study by Gürses[6], an animal model could be designed with an extremely high bilirubin exposure to investigate the effect of cortical electrical activity on regional disparity. To confirm the latter hypothesis, which has been examined in several adult human studies[27] further electrophysiological monitoring should be conducted in the BG and cortex of the same severe hyperbilirubinemia animal model.

The limitation of the present study is the lack of the complete information of the seizure, especially the long term follow up data and the therapeutic reactions.

## Conclusion

In conclusion, severe neonatal hyperbilirubinemia can selectively induce temporal and occipital lobe seizures. This is the novel finding to report of a new syndrome with the same etiology and electrophysiological features as epilepsy. The underlying mechanism should be further studied.
